# Nonthermal phase transitions in metals

**DOI:** 10.1038/s41598-020-69604-9

**Published:** 2020-07-29

**Authors:** Nikita Medvedev, Igor Milov

**Affiliations:** 1 Department of Radiation and Chemical Physics, Institute of Physics, Czech Academy of Sciences, Na Slovance 2, 182 21 Prague 8, Czech Republic; 2 Laser Plasma Department, Institute of Plasma Physics, Czech Academy of Sciences, Za Slovankou 3, 182 00 Prague 8, Czech Republic; 3 Industrial Focus Group XUV Optics, MESA+ Institute for Nanotechnology, University of Twente, Drienerlolaan 5, 7522 NB Enschede, The Netherlands

**Keywords:** Phase transitions and critical phenomena, Applied optics

## Abstract

It is well known that sufficiently thick metals irradiated with ultrafast laser pulses exhibit phonon hardening, in contrast to ultrafast nonthermal melting in covalently bonded materials. It is still an open question how finite size metals react to irradiation. We show theoretically that generally metals, under high electronic excitation, undergo nonthermal phase transitions if material expansion is allowed (e.g. in finite samples). The nonthermal phase transitions are induced via an increase of the electronic pressure which leads to metal expansion. This, in turn, destabilizes the lattice triggering a phase transition without a thermal electron-ion coupling mechanism involved. We find that hexagonal close-packed metals exhibit a diffusionless transition into a cubic phase, whereas metals with a cubic lattice melt. In contrast to covalent solids, nonthermal phase transitions in metals are not ultrafast, predicative on the lattice expansion.

## Introduction

Ultrafast deposition of high energy density into a solid target creates far-from-equilibrium states of matter with unusual properties that are not achievable in equilibrium states^1–3^. Such a material with dynamically changing properties poses fundamental difficulties for a theoretical description, as it occupies the gap in standard methods between the solid state, plasma, chemical physics, and other disciplines^4,5^.

Such energy deposition may be realized via irradiation of the target with intense femtosecond laser pulses, in particular from X-ray free-electron lasers (XFEL) such as e.g. LCLS^6^, SACLA^7^, EuXFEL^8^, etc. In an irradiated solid, energy deposition by photons excites primarily the electronic system^9^. Excited electrons transfer their energy to the ions, which may result in material modifications^10^. Upon ultrafast energy deposition into an electronic system of a material, there are two typical scenarios of material damage. Energy transfer via electron-phonon (or, more generally, electron-ion) coupling leads to heating of the atomic system, which, when exceeding the melting threshold, leads to atomic disordering. Alternatively, electronic excitation may affect the interatomic potential and destabilize the lattice without any electron-ion coupling involved. Such a mechanism is known as nonthermal melting, and has been demonstrated to take place in highly excited covalently bonded materials^1,11,12^.

Since the original work by Recoules et al.^13^, it is generally accepted in the laser-mater interaction community that metals do not melt nonthermally. Indeed, it was shown that bulk metals typically exhibit hardening of phonon modes under intense electronic excitation, in contrast to covalently bonded materials^12–14^, materials with mixed ionic-covalent bonds^15,16^, or with Peierls distortions^17^. Absence of nonthermal melting in metals was confirmed in numerous subsequent *ab-initio* simulations for various metals, see e.g. Ref.^18^. Experimental evidence supported this conclusion^19,20^.

On the other hand, softening of phonon modes in gold nanospheres was reported in Ref.^21^, and was attributed to thermal expansion of the heated clusters. In a recent experiment in Ref.^22^, phonon softening was confirmed, however, nonthermal origins of this phenomenon where indicated. In Ref.^23^, based on *ab-initio* calculations, the authors concluded that in gold, and possibly other metals, the presence of free boundaries that allow metal to expand destabilizes the atomic lattice. There is a growing evidence of effects of electronic pressure on gold expansion and damage^24–26^. Theoretical studies also demonstrated that tungsten upon electronic excitation undergoes a diffusionless (martensitic) phase transition, if allowed to change its volume^27–29^, which may be a general feature for bcc metals^30,31^.

The standard evaluation of the possibility of nonthermal melting is based on assessment of the behavior of the phonon modes upon electronic excitation^13,32^. However, such considerations overlook an effect of electronic pressure which leads to mechanical stress and may induce nonthermal damage. Thus, an assessment of a material stability upon electronic excitation must consider both effects.

In this work, we study how mechanical stress induced by electronic excitation in various metals affects the material properties and its stability. In finite size metallic systems which can expand upon electronic pressure build up, expansion of a solid leads to lattice instability resulting in phase transitions via a mechanical, rather than a thermodynamic, effect. We demonstrate the universal character of such a mechanism of damage on examples of a few materials from a wide class of metals.

## Model

To study the behavior of metals under electronic excitation, we used the hybrid code XTANT-3^10^. The code consists of a few models describing various processes in solids under irradiation with free-electron laser lights. They are interconnected within a unified model with feedbacks: each model affects another on-the-fly. A detailed description of all the involved models, their parameters and numerical details, can be found in Ref.^10^. Below we provide a brief description of it.

XTANT-3 was developed specifically to model matter under XFEL irradiation, and thus utilizes the specific conditions under such irradiation. The transient electronic distribution function upon illumination with an XFEL pulse exhibits the so-called ’bump-on-hot-tail’ shape^33,34^: it consists of a low number of electrons with high energies forming an out-of-equilibrium part, and a majority of low-energy electrons populating the bottom of the conduction band adhering to a nearly-equilibrium Fermi distribution. This allows us to treat these two fractions of electrons with individual efficient methods, as described below.

(i) Photoabsorption, resulting nonequilibrium electronic cascades, and Auger-decay of core holes, are traced by means of event-by-event Monte Carlo (MC) simulations. The MC module is essentially identical to the XCASCADE code^35^, and describes the high energy part of the electronic distribution function. It is universal, and can be applied to simulate any material. Photoabsorption cross sections are extracted from the EPICS-2017 database^36^. Note that for photon energies in the XUV/X-ray range, predominantly single photon absorption takes place, while multiphoton effects are negligible at FEL pulse parameters considered here^37^. EPICS-2017 is also used to obtain atomic parameters such as Auger decay times, ionization potentials and kinetic energies of each shell, required for impact ionization cross sections^36^. Electron impact ionization events are described with binary-encounter-Bethe (BEB) cross sections^38^. Non-equilibrium electron cascades are traced until an electron loses its energy below a certain threshold, after which it joins the low-energy fraction, modeled with a different method described below. However, the electron cascade effects are not the subject of the present study, and thus will not be discussed in detail^35^. In all simulations the laser photon energy is chosen such that only an insignificant cascading effect is induced, as shown in more detail in the Supplementary Material.
(ii) The fraction of low-energy thermalized electrons and its coupling to the atoms is modeled with rate equations including Boltzmann collision integrals^39^. It traces a transient number of electrons and their energy. They change due to excitation and income of high-energy electrons or Auger decay of core holes, both of which are described in the MC module. In our model, the low-energy electrons follow the Fermi distribution at all times. The model of matrix elements entering the collision integral developed in Ref.^39^ comes from general quantum-mechanical considerations. The Hamiltonian is provided by the tight binding model, described below. Knowing the electronic wave-functions at each time step of the simulation, the matrix elements for nonadiabatic electron transitions can be calculated for any material considered. Matrix elements then enter the Boltzmann collision integral, which allows us to calculate the electron-ion coupling. It thus depends on the transient electronic distribution, band structure, and atomic structure and motion, and is not limited to the harmonic (phononic) approximation. Energy transfer between electrons and atoms is calculated at each time step of the model.
(iii) The tight binding (TB) method in XTANT-3 was recently extended^40^ to treat metals via NRL Hamiltonian parameterization on the $\hbox {sp}^3\hbox {d}^5$ basis^41^. Diagonalization of the non-orthogonal TB Hamiltonian provides the electronic band structure, evolving with the atomic motion, and the wave functions entering the Boltzmann collision integrals within the dynamical coupling (DC) method^39^. It also provides the atomic potential energy surface at each time step, dependent on the positions of all the atoms in the simulation box, and the transient electronic distribution^42^. The interatomic potential thus changes with electronic excitation, naturally accounting for possible nonthermal phase transitions.
(iv) Atomic dynamics is traced with the molecular dynamics (MD) simulations on an evolving potential energy surface described by means of the transferable tight binding method^42^. The MD module of XTANT-3 uses the velocity Verlet algorithm, and, provided the interatomic forces are known, can work for any material. Atoms also receive additional energy from electrons, calculated within the Boltzmann collision integral described above, delivered to the atoms at each time-step via velocity scaling.

With all the other modules of XTANT-3 being universal, the critical point for calculations is the TB parameterization. The NRL parameterization used here is one of the best available transferable TB parameterizations for metals; it was rigorously tested and it proved the capability to describe various elemental solids (and some compounds) very well^41,43–45^.

XTANT-3 enables one to trace the material response to electronic excitation within or beyond the Born–Oppenheimer (BO) approximation, and compare the results to investigate the contributions of thermal and nonthermal effects^46^. We model a supercell of a metallic target with an NVE ensemble (constant number of particles, volume and energy) to simulate the bulk, or with an NPH ensemble (constant number of particles, pressure and enthalpy) within the Parrinello-Rahman method to account for a change of volume and geometry of the supercell^47^. Considering the short timescales that we study here, this simulation scheme mimics finite size systems that can change their volume. It is a known problem of the scheme that it may overestimate the expansion rate of the simulation box. However, in this work we are not interested in the exact timescales, but rather in the in-principle possibility of phase transitions triggered in a metal by electronic excitation.

To induce the electronic excitation, we assume an XUV femtosecond laser pulse (10 fs FWHM) impinging under normal incidence on the supercell at various fluences. In all simulations $t = 0$ fs corresponds to the maximum of the laser pulse intensity. As was shown before, an electronic system irradiated with such a pulse relaxes quickly into a thermalized state with a temperature elevated with respect to the atomic one^10,34,48^. The electronic cascades finish within about a femtosecond for the chosen photon energies, see the Supplementary Material. Then, we trace the material response to such an electronic excitation in ruthenium, magnesium, titanium and nickel (hcp structures), and gold, aluminum, copper and nickel (fcc structures).

## Results

**Figure 1:**
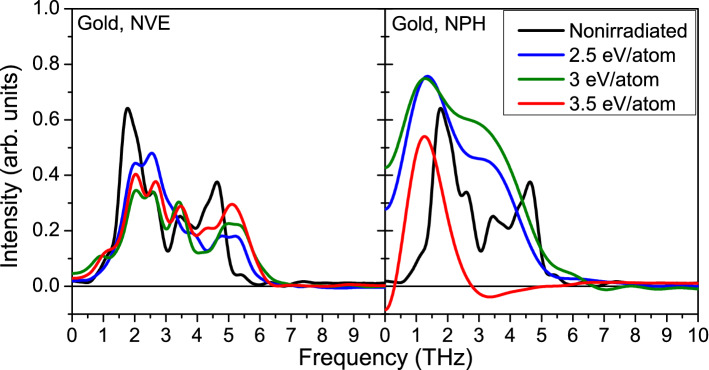
Phonon spectra in gold calculated within the NVE ensemble (left panels), and the NPH ensemble (right panels) at various deposited doses.

We start by considering an NVE ensemble (bulk) in fcc gold. Phonon spectra in gold upon energy deposition into electronic system are shown in Fig. 1 (left panel for the NVE ensemble). The spectra are calculated via the velosiy autocorrelation function averaged over 1 ps MD simulation run^10^. We can see that with the increase of the dose above $\sim 2.5$ eV/atom (and corresponding increase of the electronic temeprature), phonon spectra are shifting into higher frequencies. No phase transitions occur within the NVE simulation setup considered. This corroborates previous works which reported phonon hardening in bulk gold^13,32^, and confirms applicability of our simulation scheme to electronically excited metals.

**Figure 2:**
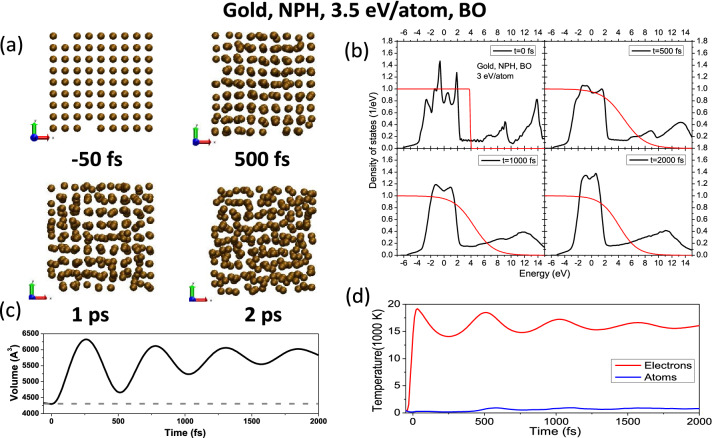
(**a**) Atomic snapshots of an NPH supercell of gold, irradiated with 3.5 eV/atom dose, modeled with XTANT-3 within the BO approximation. (**b**) Electronic DOS at corresponding instants in time; red lines depict the electron distribution function. (**c**) Evolution of the volume of the supercell (solid line); the dashed line marks the volume of the supercell at ambient conditions. (**d**) Electronic and atomic temperature evolution.

All the metals studied exhibit an increase of the electronic pressure after irradiation. In contrast to NVE, within an NPH ensemble (representing finite samples), the rise of the electronic pressure induces expansion of the supercell. The phonon modes that were hardened in an unexpanded (bulk) sample are either softened (shifted to the lower frequencies) or became unstable in an expanded (finite size) material, see the right panel in Fig. 1. The graph demonstrates the phonon softening at the doses above $\sim 2.5$ eV/atom, which supports the experiments performed with gold nanoparticles^21,22^. At the doses around 3 eV/atom and above, negative phonon frequencies appear, which is a sign of a developing lattice instability^13^. This results in nonthermal phase transition of the unstable metal at doses above this threshold. The corresponding threshold fluence for photon energies from XUV to hard X-rays can be found in the Supplementary Material.

Upon expansion, a destabilized lattice collapses into a liquid state in the case of fcc metals (Au, Al Cu, Ni), in agreement with previous *ab-initio* simulations^24–26,49^. This phase transition proceeds via the following kinetics, shown on a typical example of a gold supercell upon excitation of the electronic system with a 3.5 eV/atom dose deposited (above the nonthermal melting threshold) in Fig. 2.

We see that expansion of the supercell leads to the atomic disorder via a purely nonthermal mechanism, since the electron-ion coupling is not involved, see Fig. 2a. In this way, the atomic lattice reacts to a new potential and interatomic distance, which results into disorder at a sufficiently high dose. Expansion is crucial here – without it gold lattice remains stable, as we showed with our NVE ensemble simulation.

Upon excitation, electronic structure is also affected, as seen in Fig. 2b: the electronic density of states (DOS) smoothens due to increasing atomic disorder and narrows due to expansion. Corresponding changes in volume and both electronic and atomic temperature are shown in Figs. 2c,d.

Qualitatively, the same behaviour for Al, Cu and fcc Ni is shown in Supplementary Materials, indicating a universal nature of this damage channel for fcc elemental metals.

**Figure 3:**
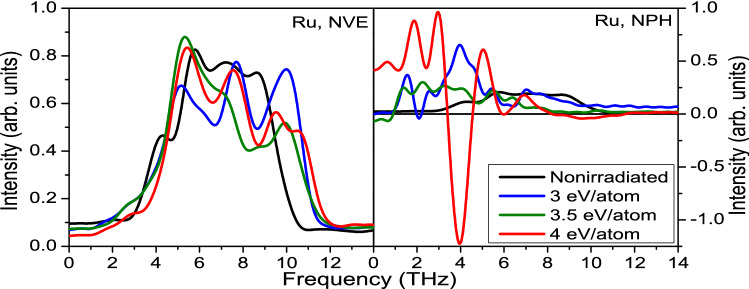
Phonon spectra in ruthenium calculated within the NVE ensemble (left panels), and the NPH ensemble (right panels) at various deposited doses.

Now let us proceed with the analysis of hcp metals. Phonon spectra in ruthenium at various irradiation doses are shown in Fig. 3. Similar to the case of gold, phonon hardening is observed within the NVE ensemble via a shift of the spectra to higher frequencies, whereas in the NPH ensemble the phonon spectra soften, and instabilities in the lattice appear at the dose of $\sim 3.5$ eV/atom and above. The calculated threshold within the NPH ensemble and the Born–Oppenheimer approximation is $\sim 3.4$ eV/atom for the nonthermal solid-solid transition in Ru. The corresponding threshold fluence is shown in the Supplementary Material.

**Figure 4:**
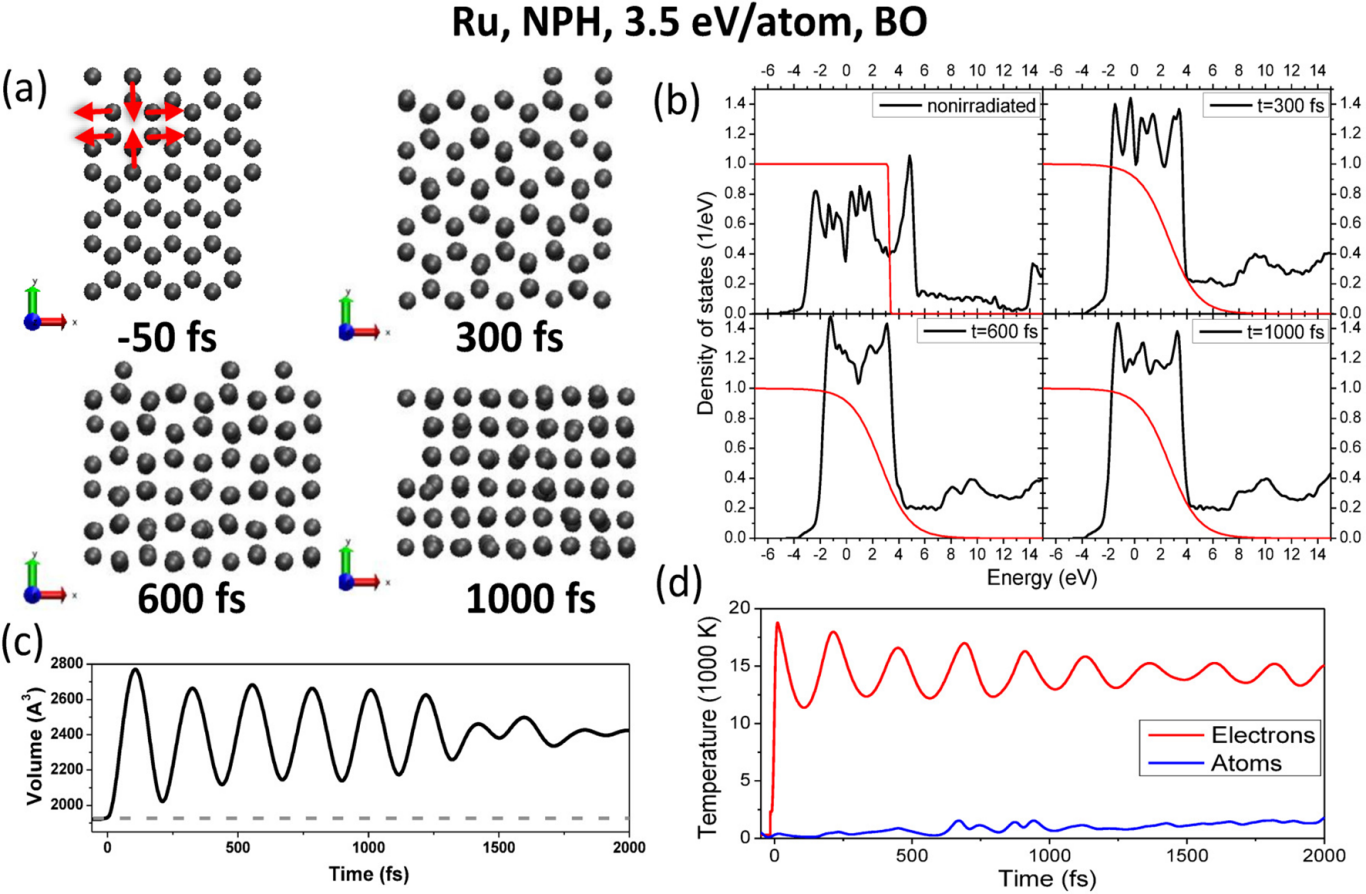
(**a**) Atomic snapshots of NPH supercell of ruthenium irradiated with 3.5 eV/atom dose, modeled with XTANT-3 within BO approximation. Red arrows indicate the direction of atomic motion leading to nonthermal diffusionless phase transition. (**b**) Electronic DOS at the corresponding time instants; thin red lines depict electron distribution function. (**c**) Evolution of the volume of the supercell; the dashed line marks the volume of the supercell at ambient conditions. (**d**) Electronic and atomic temperatures evolution.

All studied hcp elemental metals (Ru, Ti, Mg, and hcp Ni) undergo a martensitic (diffusionless) phase transition into the bcc phase at a sufficient dose if they are allowed to expand (NPH ensemble). Fig. 4 shows an example of Ru after excitation with a dose of 3.5 eV/atom. Results for the other metals can be found in the Supplementary Material.

The pathway of this transition into bcc is as follows: the lattice expands, thus stretching the in-plane structure (corresponding to optical phonons), and out-of-plane atoms in the vertices occupy the opened space (indicated by the red arrows in Fig. 4a). The electronic DOS in Ru also narrows due to material expansion as can be seen in Fig. 4b. The peaks in DOS do not completely smoothen out, in contrast to the solid-liquid phase transition in gold shown above. Additional peaks in Ru DOS appear since the atomic structure changes into a different solid state configuration.

This phase transition is faster than the melting in gold discussed above, which also agrees with the fact that the phonon spectra in the NPH simulated ruthenium acquire larger negative values (Fig. 3) than the negative values reached in the NPH simulated gold (cf. Fig. 1).

Nickel, known to exhibit phonon hardening in bulk material in both the fcc and the hcp phase^50^, within the NPH ensemble also shows nonthermal instability due to expansion, the same as other metals with respective structures: Ni in the fcc state undergoes nonthermal melting upon electronically triggered expansion, whereas hcp Ni transfers into bcc phase (see the Supplementary Material). The simulations also show that the nonthermal solid-solid phase transition (hcp $\rightarrow$ bcc) is faster than nonthermal melting of the fcc phase. Having the same atoms, Ni in both cases, we can conclude that the difference in speed of the transition is not an effect of a difference in atomic mass and inertia.

The nonthermal melting (fcc metals) or solid-solid phase transitions (hcp metals) are predicative on material expansion, and thus are not ultrafast, in contrast to the case of covalently bonded materials^10,12,51,52^. It means that the thermal nature of metal melting can not be distinguished from the nonthermal one by the mere notion of the timescales of the transition.

**Figure 5:**
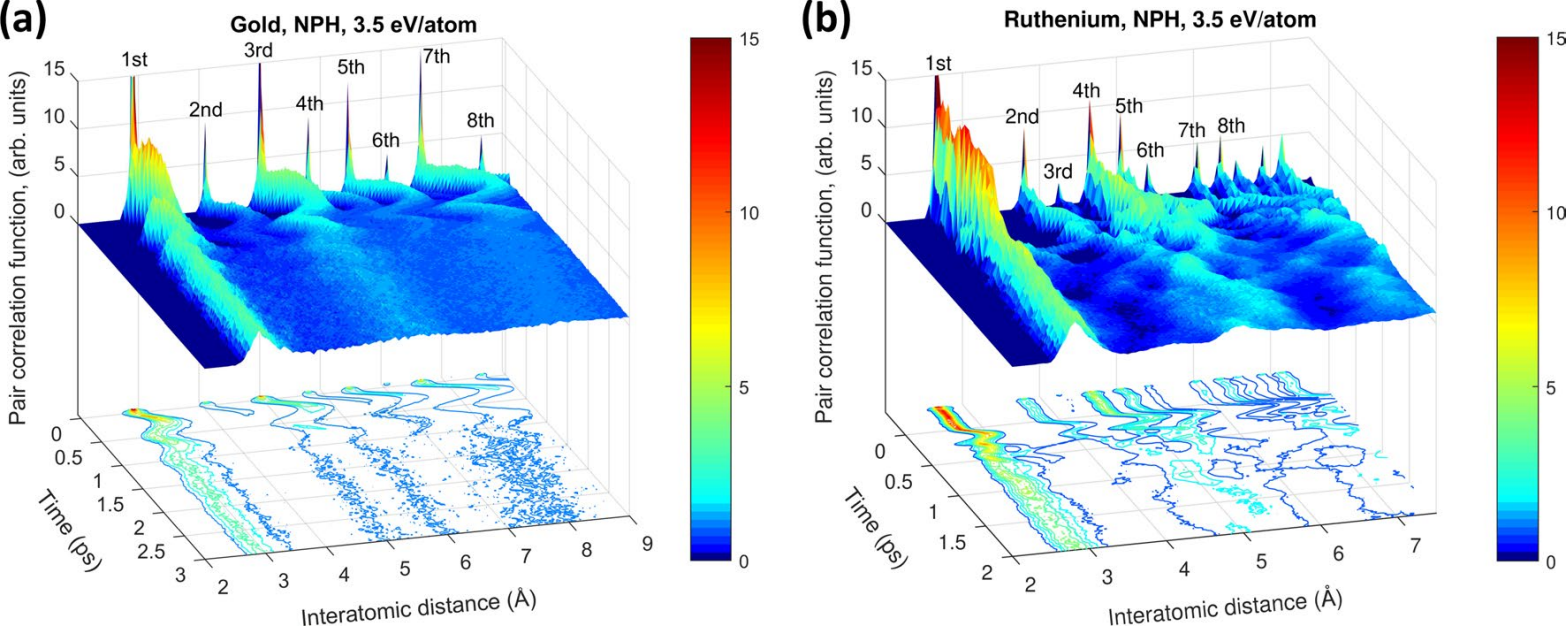
Pair correlation function evolution in (**a**) gold and (**b**) ruthenium irradiated with 3.5 eV/atom dose, modeled with NPH ensemble.

## Discussion

We compare the kinetics of nonthermal phase transitions induced in finite size gold and ruthenium, reported above. It can be traced via evolution of the pair correlation function (PCF). Fig. 5 shows that the peaks in PCF after irradiation start to oscillate due to supercell volume oscillation, after which they shift and broaden. In gold, all but the first peak disappear, corresponding to the disordered state, as is expected for liquids. In ruthenium, the PCF peaks shift to new positions corresponding to the bcc phase, and broaden but do not disappear, which is expected for a solid phase.

**Figure 6:**
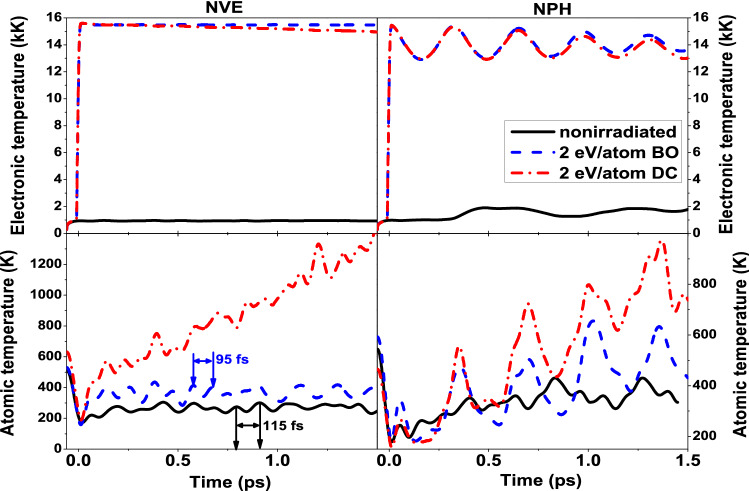
Electronic and atomic temperatures in the gold supercell within the NVE ensemble (left panels), and the NPH ensemble (right panels). The cases of nonirradiated gold, irradiated with 2 eV/atom within the BO approximation, and including dynamical electron-ion coupling (DC), are shown.

In the previous section, we demonstrated that as a response to electronic excitation, atoms gain kinetic energy (see Figs. 2d and  4d). In Fig. 6 we compare the temperatures evolution in gold for the NVE and NPH ensembles, and also with and without electron-ion coupling. The figure shows an insignificant atomic temperature increase in bulk (NVE) gold after irradiation, modeled within the BO approximation (excluding coupling), compared to the nonirradiated case. This increase occurs due to hardening of the interatomic potential as a reaction to high electronic temperature. The hardening can also be observed as the shortening of the atomic oscillation period (reflected in the atomic temperature oscillations, see Fig. 6 left panel). Large oscillations of temperatures in the NPH case (Fig. 6 right panel) are due to oscillations of the supercell volume.

This energy gain accompanies the phase transition, but does not trigger it. We emphasize that this energy increase is not a result of electron-ion coupling, since this mechanism is excluded within the BO approximation. It occurs purely due to mechanical reasons of increased pressure and expansion. This mechanism that is not related to nonadiabatic electron-ion coupling was also noted in the experiment^22^. One can see in Fig. 6 that electron-ion coupling, if included (dash-dotted curves), increases the atomic temperature additionally to the mentioned increase caused by the electronic pressure relaxation, as will be discussed below. Both, the electronic and the atomic temperature are, with the coupling included, lower in the NPH case compared to the NVE case, since a part of the absorbed energy is transiently stored in the supercell oscillations. A detailed analysis of the phase transition kinetics for gold and ruthenium at various doses around the threshold values within the Born–Oppenheimer approximation is described in the Supplementary Materials.

If we include the electron-ion coupling in our simulations, such high levels of excitations that trigger nonthermal solid-solid phase transition or melting are also sufficient to induce thermal melting after the atomic system receives a sufficient amount of energy. Significant overheating (above $\sim 1.5$ times the melting point) triggers homogeneous melting which can proceed at a picosecond timescale^53^. Thus, the nonthermally produced phase is only a transient phenomenon, which can be influenced by the thermal one. The same effect is known in covalent materials, such as silicon or diamond, where a faster nonthermal phase transition occurs before the thermal one^10,12,46,51^. However, in contrast to covalently bonded materials, in finite-size metals the nonthermal melting may not necessarily be faster than the thermal one, depending on the electron-ion coupling strength and the rate of material expansion.

As was shown in several references, both theoretical and experimental, the electron-ion coupling in gold is slow^54–56^ (as can be concluded from Fig. 6; note that the coupling parameter calculated with our approach is $\sim 10^{16}\,\hbox { W/m}^3/\hbox {K}$, in a reasonable agreement with the experimental data^54^). Thus, in samples small enough for the expansion to be faster than the significant atomic heating via the electron-ion coupling, one can expect nonthermal effects to be observable. Indeed, it seems to be the case for gold nanoclusters^22^, but not for gold films of a few tens of nm thickness^54^.

The electron-ion coupling depends on the atomic and electronic structures and motion, and thereby on parameters such as volume, pressure, electronic and atomic temperatures etc. It is possible to model it with XTANT-3. However, a detailed study of such effects is beyond the scope of this paper and will be published elsewhere^57^.

In materials where electron-ion coupling is fast, such as ruthenium^58,59^ (estimated in XTANT-3 to be $\sim 10^{17} - 10^{18}\hbox { W/m}^3/\hbox {K}$, also in a reasonable agreement with other evaluations^60^), the nonthermal phase transition into the bcc phase has a short lifetime. Shortly after that, thermal melting of the new phase starts. However, at modern X-ray free-electron laser facilities, equipped for femtosecond time-resolved diffraction experimental capabilities, one should be able to observe the nonthermal transitions^61^. Thus, we suggest that X-ray-pump X-ray-probe experiments, homogeneously heating nanoclusters and probing their structure at sub-picosecond scales, should be able to experimentally validate our predictions^62^.

Simulations of an ultrathin gold layer with open surfaces also show nonthermal melting, confirming that it is not an artifact of the chosen simulation scheme within an NPH ensemble (see the Supplementary Material). Despite the differences in atomic and electronic behavior introduced by an explicit presence of free surfaces, the qualitative conclusion is the same: nonthermal expansion of finite metallic samples destabilizes the atomic lattice. Additionally, it showed that the top single-atomic layer is ablated due to strong nonthermal expansion. It indicates that nonthermal metal ablation should also be observable upon high electronic excitation at surfaces, as was discussed e.g. in^63^. This result requires dedicated studies, which are beyond the scope of the present work.

The demonstrated mechanisms of nonthermal transitions in metals should, in principle, be valid for any photon energy. Nonthermal transitions occur when a sufficiently high energy dose is absorbed by the electronic system, independently of the particular way of energy delivery. In terms of the pulse duration, it should be sufficiently short to induce non-thermal effects before thermal ones can take over.

## Conclusions

In conclusion, with a hybrid simulation tool based on Monte Carlo, Boltzmann collision integrals and tight binding molecular dynamics, we have demonstrated the existence of both, phonon hardening (in bulk material) and nonthermal phase transitions such as melting or solid-solid transitions (in finite-size samples) elemental metals. Nonthermal transitions take place due to the increase of the electronic pressure which leads to material expansion, prominent in small samples such as e.g. nanoclusters. Predicated on material expansion, nonthermal phase transitions in metals are not ultrafast.

All fcc metals studied (Au, Cu, Al, fcc Ni) melt nonthermally, whereas all hcp metals (Ru, Ti, Mg, hcp Ni) undergo a nonthermal diffusionless transition into the bcc phase. In both cases, thermal melting also takes place on longer timescales, thus potentially allowing for observation of the transient nonthermal phases by means of, e.g., femtosecond X-ray diffraction. The similarity of the observed nonthermal phase transitions in all modelled finite-size metals suggests that the mechanisms should be universal in other metals in the same classes.

## Supplementary information

Supplementary Information.

Supplementary Information Video 1.

Supplementary Information Video 2.
