# Supplementary material for: Nonthermal phase transitions in metals
Source: Sci Rep. 2020 Jul 29;10:12775. doi: 10.1038/s41598-020-69604-9 (PMC7391778; doi:10.1038/s41598-020-69604-9)
Supplement: Supplementary file 1 — Supplementary Information. [file 41598_2020_69604_MOESM1_ESM.pdf]

Supplementary Materials for: "Nonthermal phase transitions in metals"

Nikita Medvedev[1,2] and Igor Milov[3]
1. Institute of Physics, Czech Academy of Sciences, Na Slovance 2, 182 21 Prague 8, Czech Republic
2. Institute of Plasma Physics, Czech Academy of Sciences, Za Slovankou 3, 182 00 Prague 8, Czech Republic and
3. Industrial Focus Group XUV Optics, MESA+ Institute for Nanotechnology, University of Twente, Drienerlolaan 5, 7522 NB Enschede, The Netherlands

This document contains supplemental information on the simulation parameters and data regarding nonthermal phase transitions of metallic samples that are allowed to expand (e.g. finite samples). Additional details and tests of the computational model are reported, together with the simulation results for materials that the main text refers to. The results include atomic snapshots, estimated threshold fluences, evolution of electronic structures, energies, volumes, atomic mean displacements. It shows evolution of the pair correlation functions in NPH modelled gold and ruthenium, and free energies along reaction path coordinates.

PACS numbers:

I. MODELING DETAILS

All simulations are performed with the hybrid code XTANT-3 described in the main text [1]. XTANT code has recently been extended to XTANT-3, which is capable of treating non-orthogonal Hamiltonian using Löwdin diagonalization method [2]. Tight binding (TB) Hamiltonian parameters for elemental metals used in this study are taken in the NRL form [3]. The NRL transferable TB parameterizarion has been extensively tested and studied, confirming its high reliability for description of the structural and dynamical properties of materials [4–7].

The typical supercell size in our simulation was 3x3x3 or 4x4x4 orthogonal unit cells each containing 4 atoms in the fcc or hcp symmetry. The molecular dynamics module of our code uses the velocity Verlet algorithm with a timestep of 0.1 fs for the Born-Oppenheimer (BO) simulations, and 0.02 fs for simulations with electron-ion coupling included (using dynamical coupling (DC) scheme [8]). The Parrinello-Rahman scheme was used to trace the evolution of the supercell vectors in the case of NPH atomic ensemble (constant number of particles, pressure and enthalpy) simulations with the effective supercell mass $W_{PR} = 25.5 M_{sc}$, where for elemental solids $M_{sc} = N_{at} M_{at}$ with N_{at} being a number of atoms in the supercell and M_{at} being the atomic mass of the corresponding element [9]. In the density of states (DOS) calculations we used 5x5x5 Monkhurst-Pack grid [10] for the entire supercell with 256 atoms.

To ensure stability of our simulations, we performed test runs at equilibrium conditions at the room temperature. An example for gold is shown in Fig. 1, indicating the stability of the structure, volume and temperatures, with no spontaneous phase transitions or other instabilities. This confirms that the model and parameters are reliable for further simulations.

To explicitly demonstrate phonon hardening in bulk gold, we calculated a vibrational spectrum, using the velocity autocorrelation method [11]. The results for the

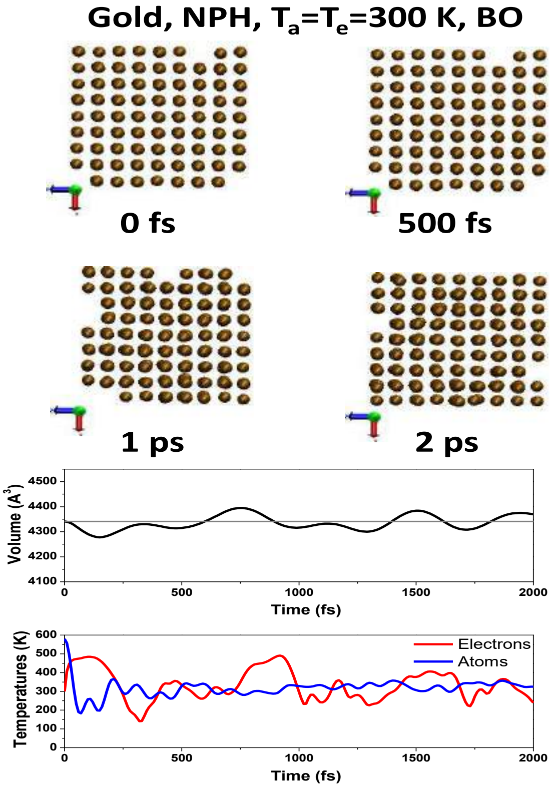

FIG. 1: Top panel: atomic snapshots of NPH supercell of gold at room temperature, modeled within the BO approximation. Middle panel: evolution of the volume of the supercell; the grey line marks the volume of the supercell at normal conditions. Bottom panel: electronic and atomic temperatures.

electronic temperatures of 300 K and 20000 K are shown in Fig. 2. A comparison with the experimental data [12] at 300 K is shown only to demonstrate a qualitative agreement in the position and width of the vibrational frequency peaks. Most importantly, our simulations show that there is a shift to higher frequencies of the entire

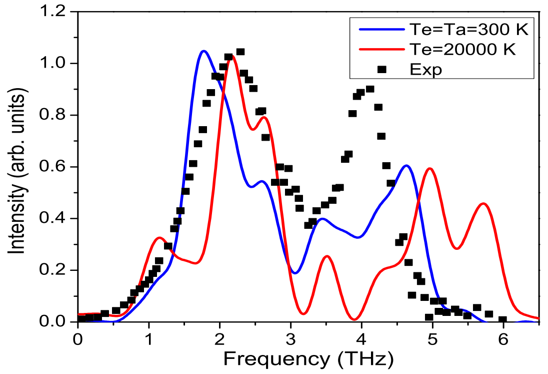

FIG. 2: Calcualted phonon spectra in gold at room temperature vs. at electronic temperature of 20 kK. Experimental points are for gold at normal conditions [12].

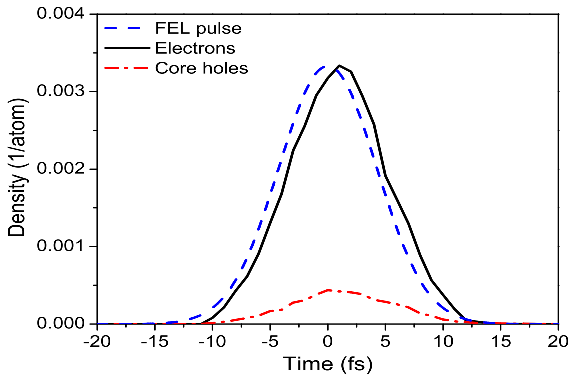

FIG. 3: Densities of excited electrons with energies above cutoff $E_{cut} = 10$ eV, and core holes in gold irradiated with 10 fs FWHM, 92 eV photon pulse.

phonon spectrum at an elevated electronic temperature with respect to the room temperature, which is a signature of the phonon hardening in bulk material.

To support our claim in the main text regarding the negligible electron cascades for the chosen irradiation parameters, we show the density of high-energy electrons and core holes in gold irradiated with 92 eV photon pulse of 10 fs FWHM duration (Fig. 3). The electrons and core holes cascades practically finish within ~ 1 fs after the pulse. Similar results are obtained for other cases considered. This confirms that our method of energy deposition into the samples, mimicking XUV/soft-X-ray FEL irradiation, does not drive electronic system into a long-lasting nonequilibrium.

The threshold dose for nonthermal melting in gold within NPH ensemble and Born-Oppenheimer approximation is $D = 3$ eV/atom; and $D = 3.4$ eV/atom for nonthermal solid-solid transition in ruthenium, as reported in the main text. We converted the absorbed threshold dose into the incoming threshold fluence F for normal incidence conditions according to the following equation [1]:

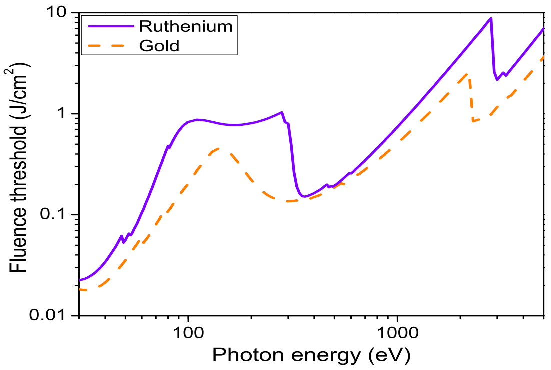

FIG. 4: Estimated damage threshold fluence in gold and ruthenium for nonthermal phase transitions simulated within the BO approximation and NPH ensemble.

$$F = \frac{D N_{at} \lambda}{(1-R)(1-\exp(-d/\lambda))} \quad (1)$$

where N_{at} is the atomic density, R is the surface reflectivity, which for the case of X-rays under normal incidence can be neglected, and λ is the photon attenuation length taken from EPICS-2017 database [13]. We are able to perform the conversion for various photon energies from XUV to hard X-rays, since the difference in cascading processes does not affect the damage threshold at normal incidence conditions [1]. We also assume that the threshold dose D is reached at the depth of the photon attenuation $d = \lambda$. The calculated threshold fluences for gold and ruthenium are shown in Fig. 4. The calculation is performed without taking into account a possible electron emission from the sample, which will depend on a particular sample geometry and FEL pulse parameters.

II. WITHIN BORN-OPPENHEIMER APPROXIMATION

A. Details of nonthermal phase transition in gold

In this section, we analyze in more detail the nonthermal melting in NPH simulated gold described in the main text. The volume oscillations at different fluences around the damage threshold, together with the mean atomic displacement, and energies in different degrees of freedom, are shown in Fig. 5.

The analysis shows that the instability occurs when the average mean atomic displacement overcomes the value of ~ 1.25 Å in fcc gold (the dose above ~ 3 eV/atom) with respect to their equilibrium positions. The initial displacements are mainly induced by the supercell volume oscillations, whose period is stretching with the increase of the fluence, due to the increase of the oscillation amplitudes. Once the supercell volume oscillations start to relax reducing their initial amplitude, it randomizes

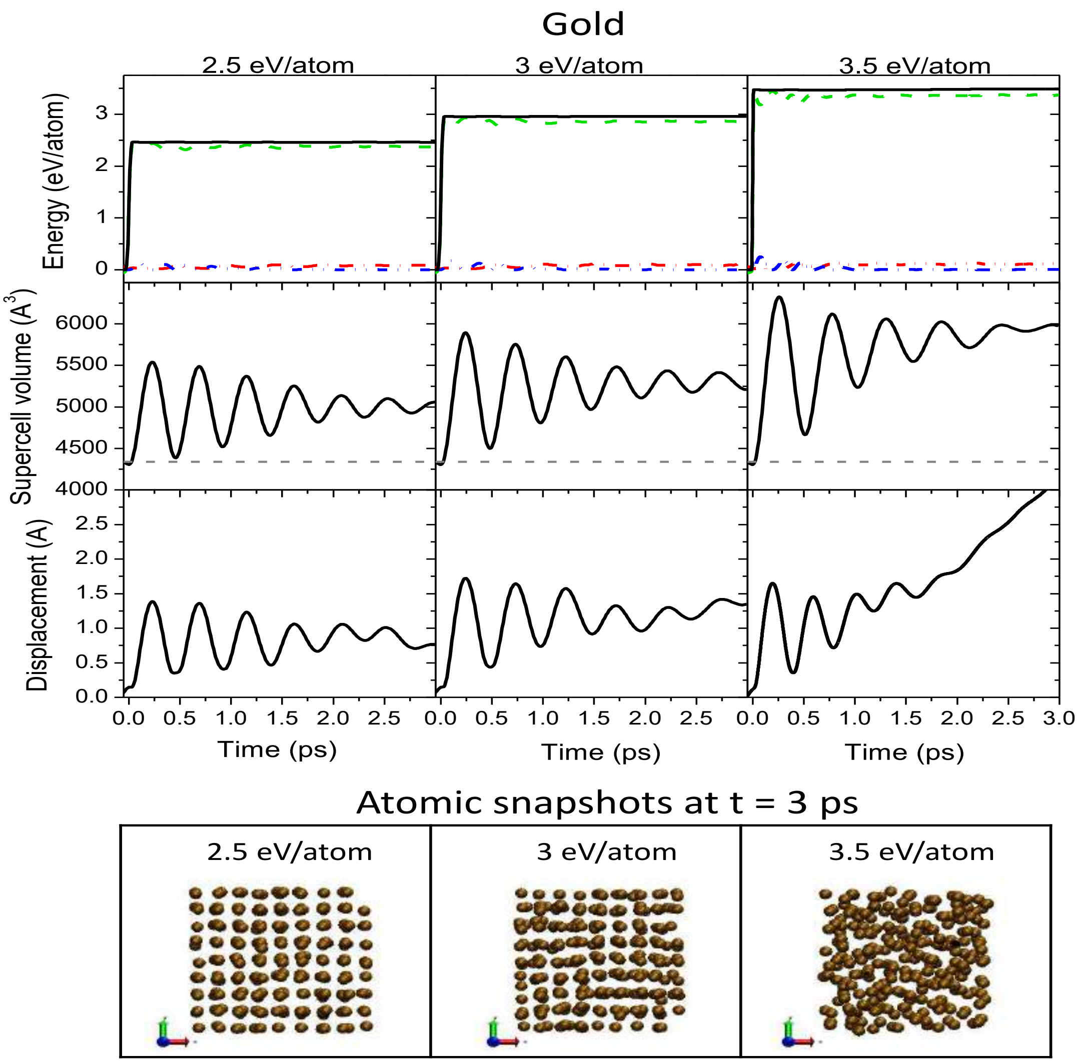

FIG. 5: Analysis of gold NPH supercell modelled within BO approximation at various fluences around the damage threshold shown in different columns. Top raw: energies in different degrees of freedom in irradiated samples. Second raw, evolution of the volume of the supercell; the dashed line marks the volume of the supercell at normal conditions. Third raw: mean atomic displacement. Bottom raw: atomic snapshots at the final time of the simulation (3 ps).

atomic motions from the initial coherent ones (see also Supplementary video file Au_BO_NPH_3.5eVatom.avi). This way, the atomic lattice reacts to the new potential and interatomic distance, which results into disorder at a sufficiently high dose in gold.

The final atomic snapshots (at $t = 3$ ps) are shown in the bottom panel of Fig. 5. One can see that the dose of 3 eV/atom is sufficient to induce disorder in the gold lattice. The degree of disorder increases further with the increasing dose. The final state after 3.5 eV/atom dose deposition is liquid, as can be seen by contineous increase of the mean atomic displacement.

The Fig. 5 also shows the energies stored in different degrees of freedom: the atomic potential energy, kinetic energy, and the energy of the supercell oscillations, together with the total energy in the system which is conserved (apart from the initial instant of the energy deposition by the laser pulse). The total energy is conserved due to microcanonical ensemble we use (the precision of energy conservation is defined by the relatively large simulation boxes and long timescales modelled, which limited the choice of the timestep). Initially, during interaction with the laser pulse, the deposited energy splits into the potential energy of the atoms (a major part), and the energy of the supercell oscillations. The atomic kinetic energy is almost unchanged. As the supercell oscillations stabilize, their amplitude reduces, and the corresponding energy loss transfers into the gain in the atomic kinetic energy: kinetic energy becomes larger than the supercell energy. The kinetic energy at the near threshold dose is sufficient to overcome the modified barriers for atomic disorder in case of gold (or a transition into bcc phase

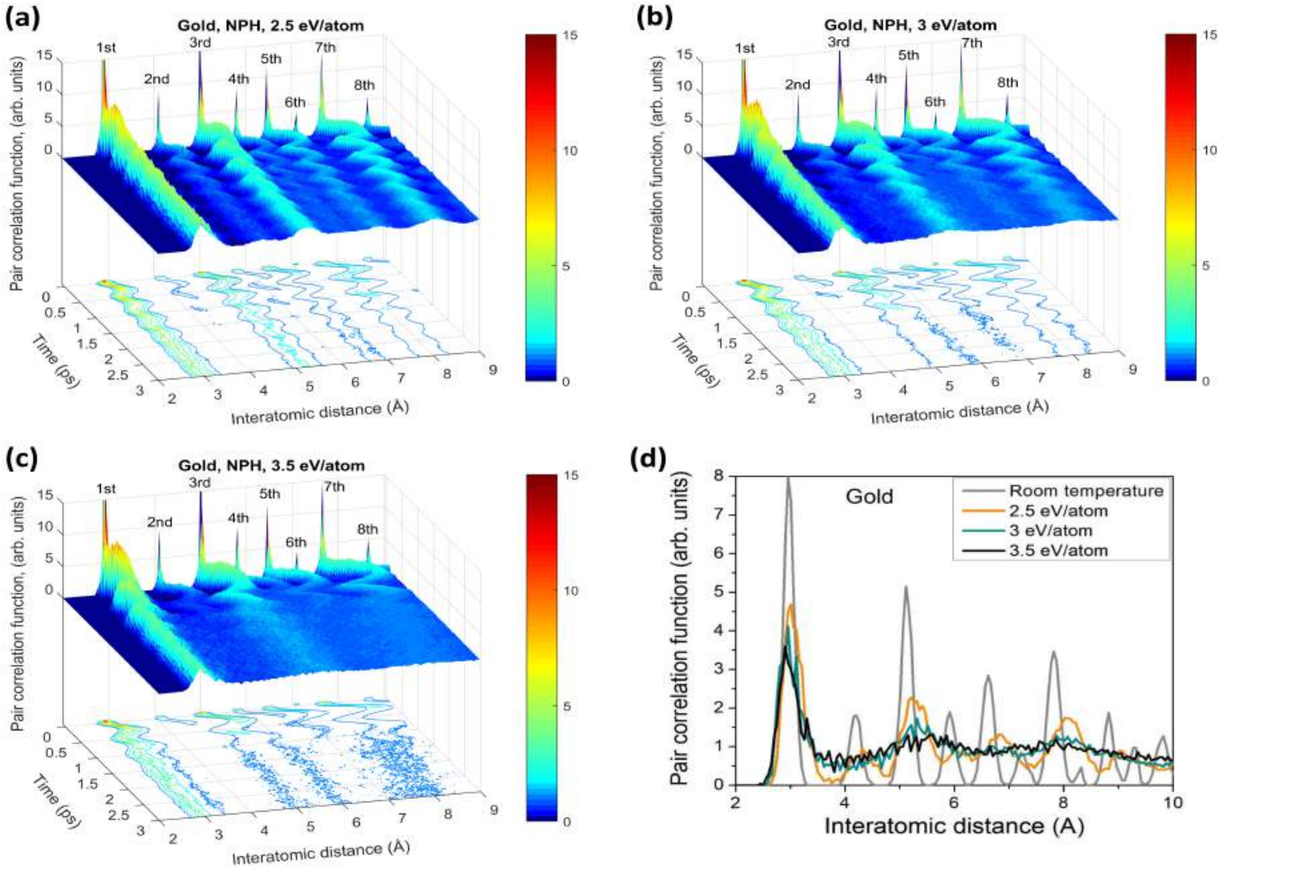

FIG. 6: Pair correlation function (PCF) in supercell of gold. (a) Evolution of PCF aver time after 2.5 eV/atom dose deposition. (b) PCF for 3 eV/atom dose. (c) PCF for 3.5 eV/atom dose. (d) PCF at final time instants at the corresponding doses.

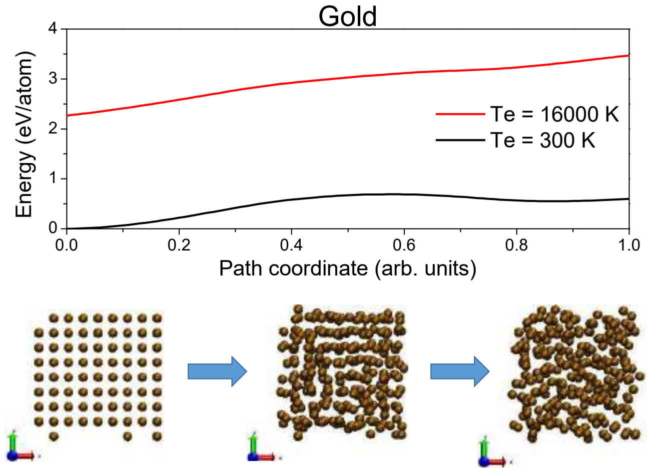

FIG. 7: Top panel: free energies in gold along the path coordinate from perfect fcc lattice to the melted state for electronic temepreatures of 300 K and 16000 K. Bottom panel: Snapshots of the atomic lattice at the initial, middle, and final states.

for ruthenium, see below Fig. 11).

Disorder in gold can be traced via a fast disappearance of the even peaks (2nd, 4th, etc.) in the atomic pair correlation function (PCF), whereas odd (1st, 3rd, etc.) peaks persist for longer times, see Fig. 6. One can see in the PCF that the second and fourth nearest neighbors lose their order, whereas the first nearest neighbor peak remains sharp, and the third one broadens but is still present at above-threshold dose. It is also clear that the long order disappears much faster than the short order. Such a behavior is expected during melting.

We calculated the free energy of gold supercell along the path coordinate from the ideal fcc lattice into the melted state corresponding to the material irradiated with 3.5 eV/atom shown in Fig. 5 (bottom right panel). Figure 7 shows the free energies calculated at the electronic temperatures of 300 K and 16000 K. The latter temperature is chosen corresponding to the final temperatures reached in the MD simulation.

The results suggest that the energy barrier between the two phases in the unexcited material (at T_e = 300 K) is $\sim$ 1 eV/atom. The minimum on the right can be considered as a possible amorphous metastable state, although one has to note here that this is only one of the various possible paths.

At elevated electron temperature T_e = 16000 K, the minimum disappears, indicating unstable (melted)

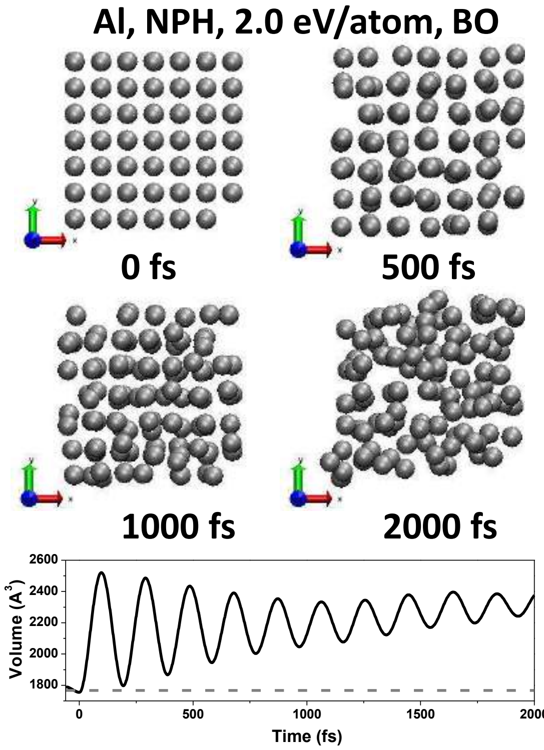

FIG. 8: Top panel: atomic snapshots of NPH supercell of Al irradiated with a laser pulse of 50 eV photon energy, 10 fs FWHM, 2 eV/atom absorbed dose, modeled within the BO approximation. Bottom panel: evolution of the volume of the supercell; the dashed line marks the volume of the supercell at normal conditions.

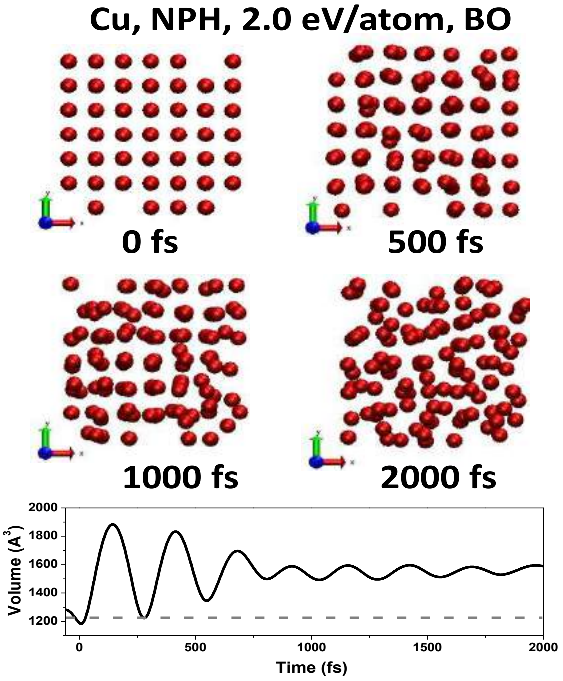

FIG. 9: Top panel: atomic snapshots of NPH supercell of Cu irradiated with a laser pulse of 92 eV photon energy, 10 fs FWHM, 2 eV/atom absorbed dose, modeled within the BO approximation. Bottom panel: evolution of the volume of the supercell; the dashed line marks the volume of the supercell at normal conditions.

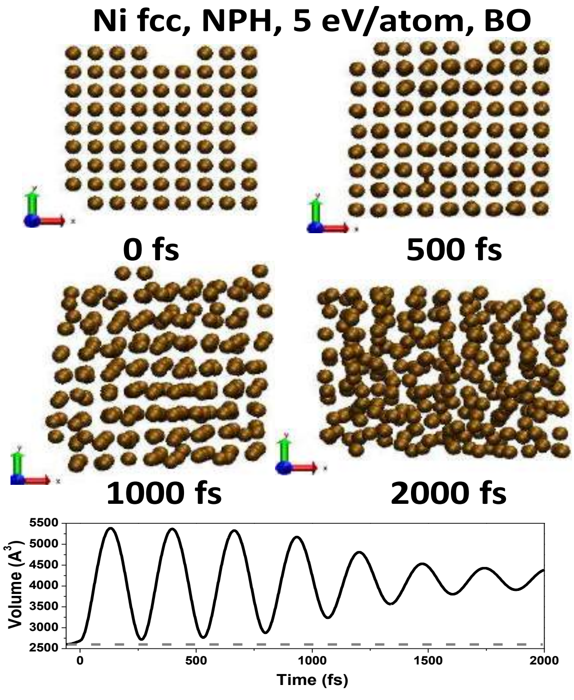

FIG. 10: Top panel: atomic snapshots of NPH supercell of Ni in the fcc phase irradiated with a laser pulse of 50 eV photon energy, 10 fs FWHM, 5 eV/atom absorbed dose, modeled within the BO approximation. Bottom panel: evolution of the volume of the supercell; the dashed line marks the volume of the supercell at normal conditions.

phase. The presented results also suggest that fast cooling of the electronic system in this state could potentially freeze gold in the disordered phase.

B. Other fcc metals

We show the results of XTANT-3 simulations for fcc metals within the Born-Oppenheimer approximation. The irradiation parameters in each particular case (throughout the whole work) are chosen to avoid a significant electron cascading and to induce a detectable phase transition. Atomic snapshots and a supercell volume evolution of aluminum are shown in Fig. 8. The NRL Hamiltonian [6] was augmented with a strong short-range repulsive force to prevent atoms from coming too close to each other after excitation [11]. Nonthermal melting due to material expansion caused by an increase of the electronic pressure can be identified, together with the supercell volume increase. Similar results are obtained for copper and fcc nickel, Figs. 9 and 10, respectively.

The results suggest that elemental fcc metals disorder upon electronic excitation if given an opportunity to relax the built-up electronic pressure via expansion.

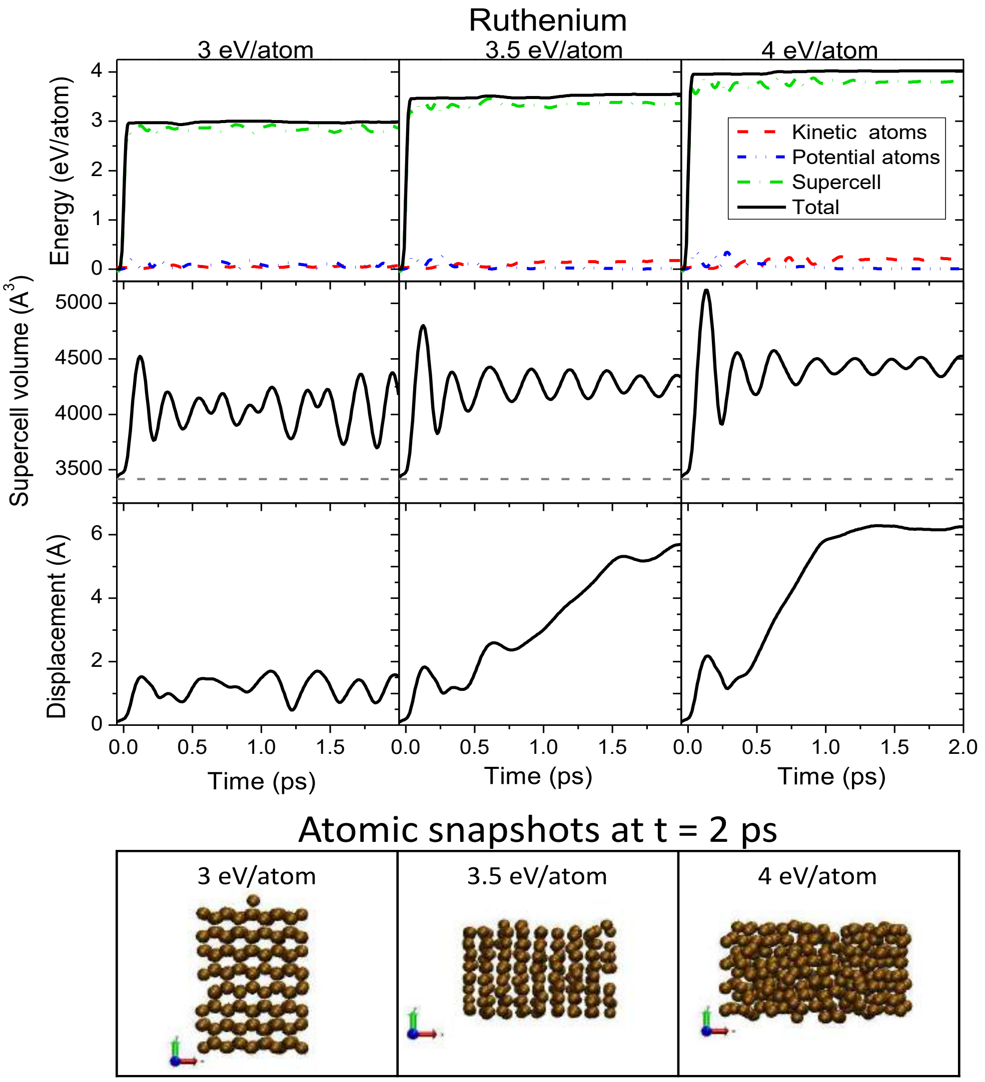

FIG. 11: Analysis of ruthenium NPH supercell modelled within BO approximation at various fluences around the damage threshold shown in different columns. Top raw: energies in different degrees of freedom in irradiated samples. Second raw, evolution of the volume of the supercell; the dashed line marks the volume of the supercell at normal conditions. Third raw: mean atomic displacement. Bottom raw: atomic snapshots at the final time of the simulation (2 ps).

C. Details of nonthermal phase transition in ruthenium

The energies, supercell volume, and mean atomic displacements in ruthenium are shown in Fig. 11. They behave qualitatively similar to the case of gold discussed above. The main difference is that at the dose of 3.5 eV and higher, the atomic displacement increases significantly compared to the normal oscillations, and saturates at the new value, corresponding to the new bcc structure formed (whereas in gold there was a continuous growth due to liquid phase formation).

In ruthenium, by the time of ~ 1 ps, the peaks in the PCF are shifted and broadened as seen in Fig. 12, but did not disappear into a diffuse background, which is consistent with the bcc solid phase formation. The PCF evolves coherently at first due to supercell volume oscillations, but once the oscillations stabilize with a small amplitude, atoms rearrange inside of the cell (see an example in the supplementary video file Ru_BO_NPH_3.5eVatom.avi). Note that at the near-threshold dose of 3.5 eV/atom the PCF peaks of the new phase are sharper than those at higher dose of 4 eV/atom, indicating wild atomic oscillations close to disorder/melting at the highest dose. The onset of disorder can also be seen in the atomic snapshot in Fig. 11.

Figure 13 shows the free energies in ruthenium along path coordinate from hcp to formed bcc state calculated at the electronic temperatures of 300 K and 15000 K. The latter temperature is chosen corresponding to the final temperatures reached in the MD simulation at 3.5 eV/atom. The results show that the energy barrier be-

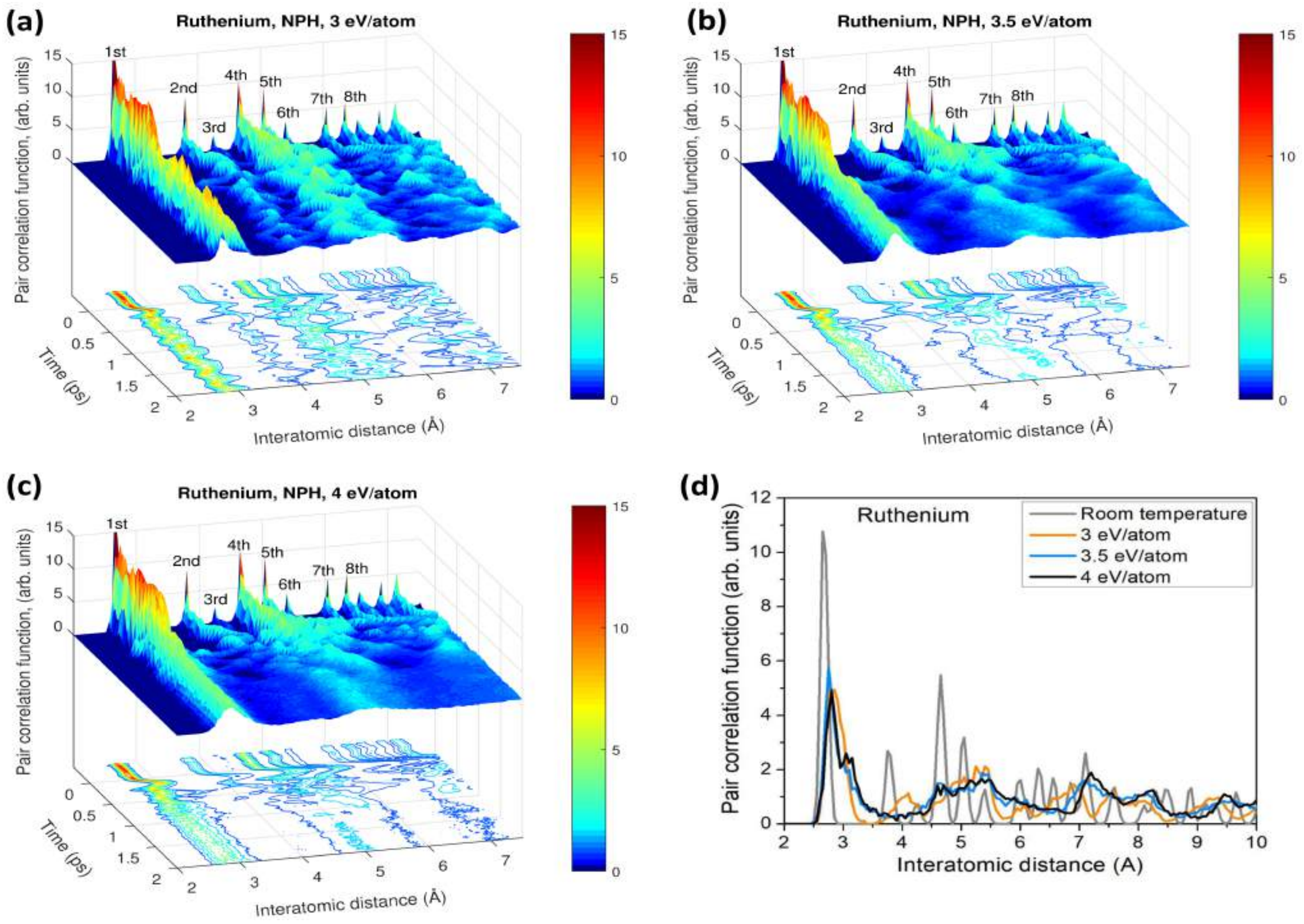

FIG. 12: Pair correlation function (PCF) in supercell of ruthenium. (a) Evolution of PCF aver time after 3 eV/atom dose deposition. (b) PCF for 3.5 eV/atom dose. (c) PCF for 4 eV/atom dose. (d) PCF at final time instants at the corresponding doses.

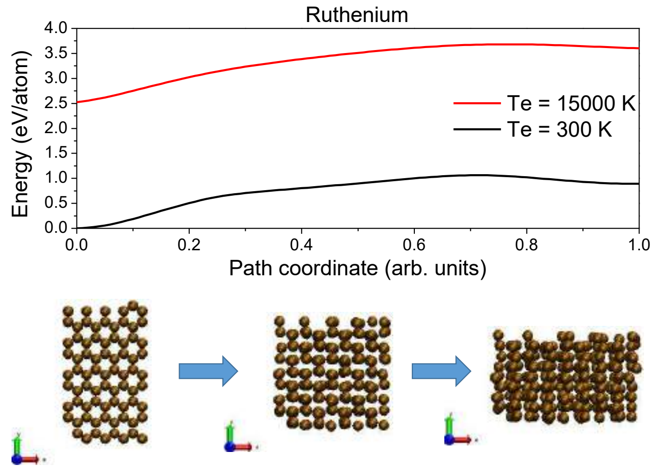

FIG. 13: Top panel: free energies in ruthenium along the path coordinate from perfect hcp lattice to the formed bcc phase for electronic temepreatures of 300 K and 15000 K. Bottom panel: Corresponding snapshots of the atomic lattice at the initial, middle, and final states.

tween the two phases is $\sim$ 1.25 eV/atom for both, electrons at room temperature and at T_e = 15000 K. The deposited dose of 3.4 eV/atom and above is sufficient to overcome the barrier even for atoms at nearly room temperature, inducing the nonthermal solid-solid phase transition. The height of the barrier for the reverse transition from bcc to hcp phase is only $\sim$ 0.2 eV/atom, which means that heating of the atomic system will melt the formed bcc phase. More on this point will be discussed below in the section III.

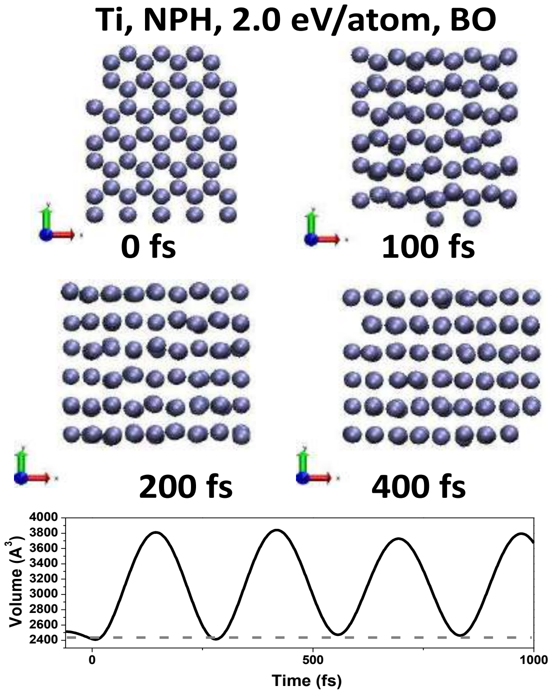

FIG. 14: Top panel: atomic snapshots of NPH supercell of Ti irradiated with a laser pulse of 92 eV photon energy, 10 fs FWHM, 2 eV/atom absorbed dose, modeled within the BO approximation. Bottom panel: evolution of the volume of the supercell; the dashed line marks the volume of the supercell at normal conditions.

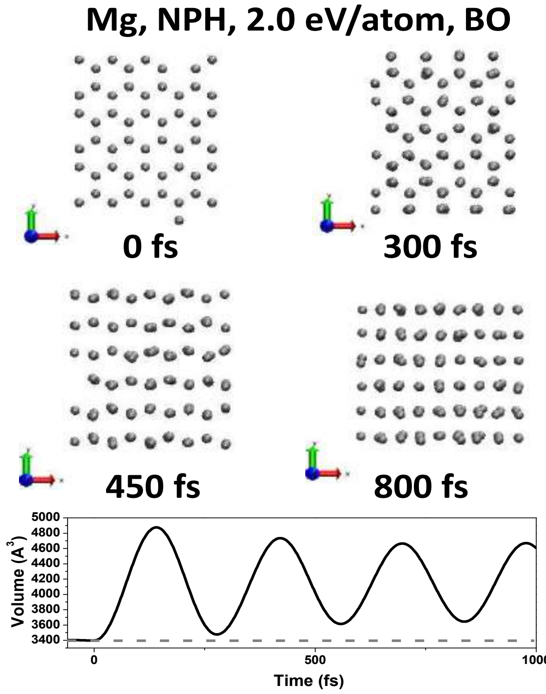

FIG. 15: Top panel: atomic snapshots of NPH supercell of Mg irradiated with a laser pulse of 50 eV photon energy, 10 fs FWHM, 2 eV/atom absorbed dose, modeled within the BO approximation. Bottom panel: evolution of the volume of the supercell; the dashed line marks the volume of the supercell at normal conditions.

D. Other hcp metals

Here we show simulation results for hcp metals. Atomic snapshots in titanium supercell shown in Fig. 14 demonstrate nonthermal martinsitic (diffusionless) phase transition due to material expansion into bcc phase, qualitatively the same as in Ru reported in the main text. Similar results are also obtained for magnesium and hcp nickel, Figs. 15 and 16, respectively.

On the example of nickel we can compare solid-liquid (nonthermal melting of the fcc phase) and solid-solid (hcp to bcc) phase transitions for a material with an equal atomic mass. The results show that the nonthermal melting takes longer time than the solid-solid diffusionless phase transition.

The results strongly indicate that the diffusionless solid-solid phase transition is a universal response of hcp metals to electronic excitation and consequent relaxation of the electronic pressure within NPH ensemble simulations.

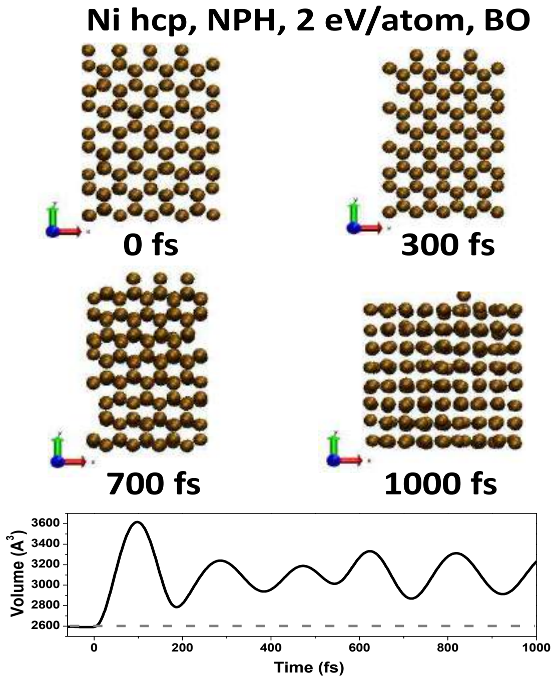

FIG. 16: Top panel: atomic snapshots of NPH supercell of Ni in the hcp phase irradiated with a laser pulse of 50 eV photon energy, 10 fs FWHM, 2 eV/atom absorbed dose, modeled within the BO approximation. Bottom panel: evolution of the volume of the supercell; the dashed line marks the volume of the supercell at normal conditions.

III. BEYOND BORN-OPPENHEIMER APPROXIMATION

In this section we go beyond the Born-Oppenheimer approximation and take into account the electron-ion en-

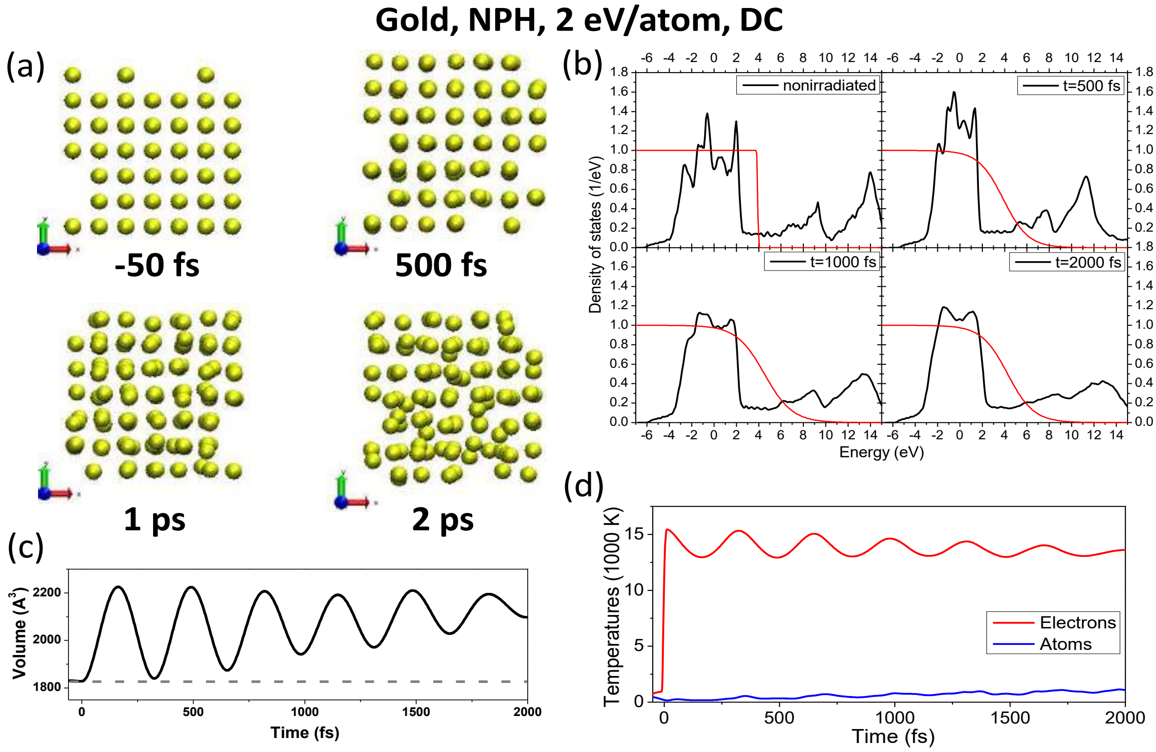

FIG. 17: (a) Atomic snapshots of NPH supercell of Au irradiated with a laser pulse of 92 eV photon energy, 10 fs FWHM, 2 eV/atom absorbed dose, modeled with electron-ion coupling included. (b) Electronic DOS at the corresponding time instants; thin red lines depict electron distribution function. (c) Evolution of the volume of the supercell; the dashed line marks the volume of the supercell at ambient conditions. (d) Electronic and atomic temperatures evolution.

ergy exchange mechanism. We include it within the dynamical coupling (DC) calculations of the probabilities of electronic transition induced by atomic motion [8]. The collision integral is written without the assumption of the Fermi's golden rule and phononic approximation, and thus is applicable at any timescale, including the femtosecond range [8]. A detailed study of the coupling parameters within our developed approach can be found in Ref. [14].

An example of gold irradiated with an FEL pulse depositing 2 eV/atom dose is shown in Fig. 17. The figure shows expansion of the supercell induced by the electronic pressure which results in melting. The electron-ion coupling in gold at such a deposited dose is too slow for atoms to reach the melting point at the timescale of occurring disorder, as seen in Fig. 17d. The electron-ion coupling does not induce the thermal melting during the calculation time, however, it reduces the threshold dose of nonthermally induced melting. We can also see in the figure that the electronic density of states (DOS) is affected: during the expansion of the supercell, DOS narrows (see Fig. 17b at 500 fs). Later, due to the atomic heating, it broadens again to a larger width than in the simulation within the BO approximation (cf. the main text). When atomic disorder takes place, sharp peaks disappear from the DOS.

A simulation of ruthenium with the electron-ion coupling included shows that the nonthermally produced bcc phase starts to melt thermally due to the heating of the atomic system by electrons, see Fig. 18. Heating also affects the electronic structure, as seen in panel b of the same figure, resulting in smoothing of the peaks. Nonetheless, some peaks are still present, resembling the bcc DOS, cf. the main text. Under such conditions, ruthenium undergoes a martinsitic phase transition. However, the electron-ion coupling in Ru is very strong (as seen in the temperatures equilibration process shown in panel d), which results in disorder starting to take place within a picosecond [15]. Due to the competing phase transitions: solid-solid into bcc via nonthermal channel, and solid-liquid due to thermal melting, the atomic positions do not align into bcc phase as perfectly as within the Born-Oppenheimer approximation (cf. Fig.4 in the main text). This is also reflected in the electronic structure as broadening and disappearance of the peaks.

By the time of 1 ps, the supercell is completely disordered. The transient bcc phase lasts only until the atomic heating melts the new phase thermally. Thus, we expect that nonthermal phase transitions in small nanoclusters of ruthenium, which could expand sufficiently fast to outrun the thermal damage, would be observable in experiments. Alternatively, fast and efficient cooling of the target could potentially freeze the forming bcc phase.

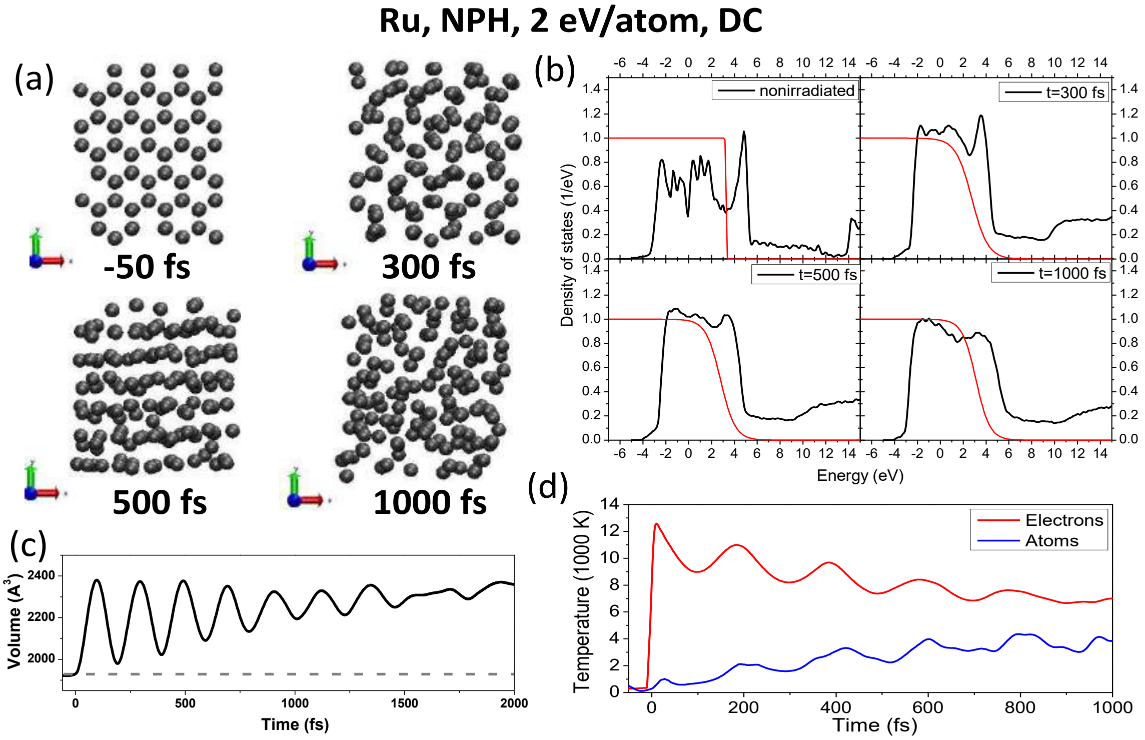

FIG. 18: (a) Atomic snapshots of NPH supercell of Ru irradiated with a laser pulse of 92 eV photon energy, 10 fs FWHM, 2 eV/atom absorbed dose, modeled with electron-ion coupling included. (b) Electronic DOS at the corresponding time instants; thin red lines depict electron distribution function. (c) Evolution of the volume of the supercell; the dashed line marks the volume of the supercell at ambient conditions. (d) Electronic and atomic temperatures evolution.

For that purpose, one could employ laser irradiation conditions such that the electrons are heated only in a thin near-surface layer of a Ru target (e.g. employing the photon wavelength corresponding to the plasmon energy thereby reducing the skin depth, or using grazing incident XUV or X-ray pulses). Efficient energy transport in this case could cool down the system sufficiently fast [16].

IV. ULTRATHIN LAYERS

We modeled an ultrathin layer of material in order to analyze a response of a free surface and to cross check the results against those obtained within the NPH ensemble (which, although allows the supercell to expand, does not include free surfaces). The main difference is that, in contrast to the case of the NPH ensemble, atoms can escape from a sample with free surfaces.

We modeled a thin layer of gold with open surfaces along Z-axis and periodic boundary conditions along X and Y. We used the BO approximation scheme for this simulation. Fig. 19 shows that upon electronic pressure build up due to electronic excitation, an atomic layer of gold can be ejected, thus producing a nonthermal ablation. The remaining layer exhibits disordering, thus confirming that the simulation scheme employed does not produce significant artefacts. The disorder is not as prominent as within the NPH supercell, which is probably due to ablative cooling: the atoms emitted from the surface take some energy with them out of the remaining layer of the material.

[1] N. Medvedev, V. Tkachenko, V. Lipp, Z. Li, and B. Ziaja, "Various damage mechanisms in carbon and silicon materials under femtosecond X-ray irradiation," *4open*, vol. 1, p. 3, 2018.

[2] A. Szabo and N. S. Ostlund, *Modern quantum chemistry: introduction to advanced electronic structure theory*. Dover Publications, 1996.

[3] The NRL parameters files were found available online at: http://esd.cos.gmu.edu/tb/tbp.html

[4] M. J. Mehl and D. A. Papaconstantopoulos, "Applications of a tight-binding total-energy method for transition and noble metals: Elastic constants, vacancies, and surfaces of monatomic metals," *Physical Review B*, vol. 54, pp. 4519–4530, aug 1996.

[5] M. J. Mehl and D. A. Papaconstantopoulos, "Tight-binding study of high-pressure phase transitions in tita-

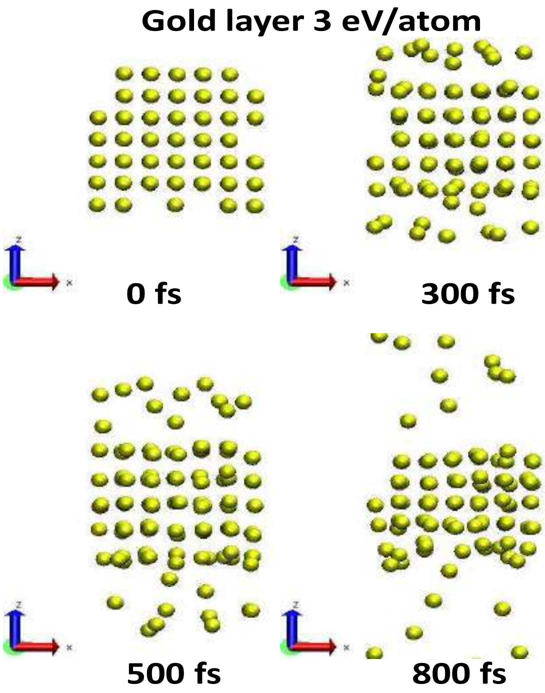

FIG. 19: Atomic snapshots of an ultrathin layer of Au irradiated with a laser pulse of 92 eV photon energy, 10 fs FWHM, 2 eV/atom absorbed dose, modeled within the BO approximation.

nium: Alpha to omega and beyond," *Europhysics Letters (EPL)*, vol. 60, pp. 248–254, oct 2002.

[6] D. A. Papaconstantopoulos and M. J. Mehl, "The Slater-Koster tight-binding method: a computationally efficient and accurate approach," *Journal of Physics: Condensed Matter*, vol. 15, pp. R413–R440, mar 2003.

[7] S. Silayi, D. Papaconstantopoulos, and M. Mehl, "A tight-binding molecular dynamics study of the noble metals Cu, Ag and Au," *Computational Materials Science*, vol. 146, pp. 278–286, apr 2018.

[8] N. Medvedev, Z. Li, V. Tkachenko, and B. Ziaja, "Electron-ion coupling in semiconductors beyond Fermi's golden rule," *Physical Review B*, vol. 95, p. 014309, jan 2017.

[9] N. Medvedev, H. O. Jeschke, and B. Ziaja, "Nonthermal phase transitions in semiconductors induced by a femtosecond extreme ultraviolet laser pulse," *New Journal of Physics*, vol. 15, p. 015016, jan 2013.

[10] H. J. Monkhorst and J. D. Pack, "Special points for Brillouin-zone integrations," *Physical Review B*, vol. 13, pp. 5188–5192, jun 1976.

[11] N. Medvedev, "Modeling warm dense matter formation within tight binding approximation," in *Optics Damage and Materials Processing by EUV/X-ray Radiation VII* (L. Juha, S. Bajt, and S. Guizard, eds.), vol. 11035, p. 25, SPIE, apr 2019.

[12] J. A. Muñoz, M. S. Lucas, L. Mauger, I. Halevy, J. Horwath, S. L. Semiatin, Y. Xiao, P. Chow, M. B. Stone, D. L. Abernathy, and B. Fultz, "Electronic structure and vibrational entropies of fcc Au-Fe alloys," *Physical Review B*, vol. 87, p. 014301, jan 2013.

[13] D. E. Cullen, "A Survey of Atomic Binding Energies for use in EPICS2017," tech. rep., Nuclear Data Section, International Atomic Energy Agency, Vienna, 2018.

[14] N. Medvedev and I. Milov, "Electron-phonon coupling in metals at high electronic temperatures," 2020. https://arxiv.org/abs/2005.05186

[15] I. Milov *et al.*, "Mechanism of single-shot damage of Ru thin films irradiated by femtosecond extreme UV free-electron laser," *Optics Express*, vol. 26, p. 19665, jul 2018.

[16] I. Milov, V. Lipp, N. Medvedev, I. A. Makhotkin, E. Louis, and F. Bijkerk, "Modeling of XUV-induced damage in Ru films: the role of model parameters," *JOSA B*, vol. 35, no. 10, pp. B43–B53, 2018.
